# Meniscal stress biomechanics in Tai Chi’s Brush knee and Twist Step for elderly

**DOI:** 10.3389/fbioe.2025.1620228

**Published:** 2025-08-22

**Authors:** Fengzhen Chen, Huan Chen, Sheng Chen, Jiaqiu Lin

**Affiliations:** ^1^ School of Physical Education and Sport Science, Fujian Normal University, Fuzhou, Fujian, China; ^2^ School of Physical Education, Minjiang University, Fuzhou, Fujian, China; ^3^ The Third People’s Hospital Affiliated to Fujian University of Traditional Chinese Medicine, Fuzhou City, Fujian, China

**Keywords:** Tai Chi, meniscus, elderly, biomechanics, brush knee and twist step

## Abstract

**Objective:**

This study investigates the biomechanical effects of long-term Tai Chi practice on the knee meniscus through biomechanical experimentation and finite element simulation, focusing on practitioners performing Knee Brushing and Twisting Step. The findings aim to establish scientific guidelines for optimizing exercise protocols in middle-aged and elderly populations.

**Methods:**

Twenty male middle-aged and elderly practitioners were recruited, divided into a Beginner Group (BG: n = 10), and an Experienced Group (EG: n = 10). Kinematic and kinetic data during Knee performance of Brushing and Twisting Step were collected using synchronized three-dimensional infrared motion capture and force platforms. A finite element model was developed and validated based on knee CT and MRI imaging data from a representative participant with average anthropometric measurements. The acquired kinematic and kinetic data were applied as boundary conditions and loading inputs in finite element analysis software to simulate the knee joint contact stress distribution during movement execution.

**Results:**

(1) The Experienced Group demonstrated significantly greater knee flexion angles compared to the Beginner Group across all movement phases (*P* < 0.01), while exhibiting significantly lower varus-valgus and internal-external rotation angles (*P* < 0.01). (2) The Experienced Group exhibited phase-specific peak contact stress distribution: predominantly on the lateral meniscus during (left) double-support, (left) swing, (left) single-support, and terminal (right) swing phases, shifting to the medial meniscus during (right) double-support, initial (right) swing, and (right) single-support phases. In contrast, the Beginner Group demonstrated consistent lateral meniscus stress concentration across all phases except during the (right) swing phase.

**Conclusion:**

Long-term practice of Tai Chi optimizes the distribution of stress across the knee joints, effectively reducing localized stress concentrations and the associated risks of meniscal injuries. For novice practitioners, it is crucial to emphasize precision in movement and adherence to technical standards to prevent knee injuries that may arise from improper biomechanical loading patterns.

## 1 Introduction

Knee joint health is critical for the daily activities and quality of life of middle-aged and elderly individuals. However, with advancing age, the risk of meniscal degeneration and injury rises significantly, becoming a primary cause of knee joint diseases in older adults (Jang et al., 2021). The meniscus, situated between the femoral condyles and the tibial plateau (Pillai et al., 2018), is a wedge-shaped fibrocartilage disc that serves to protect articular cartilage, provide joint lubrication, and enhance joint stability (Murakami et al., 2019). It reduces contact stress and increases the contact area (Kettelkamp and Jacobs, 1972; Walker and Erkiuan, 1975). Once damaged, it disrupts normal knee joint kinematics, increases peak contact stress, and leads to early degenerative changes in the knee joint, potentially resulting in complications such as knee osteoarthritis (Papalia et al., 2013; Shi et al., 2018; Sung et al., 2013).

Tai Chi, characterized by its integration of hardness and softness, movement and stillness, and coordination of internal and external elements, is a low-intensity aerobic exercise. Research shows that trunk stability training enhances pelvic and spinal control, providing a stable foundation for lower-limb movement. Improved core stability facilitates more effective coordination between the upper and lower body, resulting in better activation of the leg muscles and more efficient force transfer (Şahin et al., 2015). Similarly, practicing Tai Chi requires coordination between the upper and lower limbs to transmit force more effectively. These mechanisms may reduce abnormal joint loads and promote healthier biomechanics in the knee joint, which is important for older adults. Furthermore, long-term Tai Chi practice enhances lower limb muscle strength, improves knee joint stability, and increases functional mobility. It is particularly effective at alleviating knee pain (Kelley et al., 2022). Due to its safety and effectiveness, Tai Chi is recommended by domestic and international rehabilitation guidelines as a suitable long-term aerobic exercise therapy for middle-aged and elderly individuals (Brosseau et al., 2017; Ye and Liu, 2023).

Tai Chi encompasses a wide variety of footwork, with the advancing step being the most fundamental and frequently utilized movement. This step is a key element in the process of learning and mastering Tai Chi (Liu et al., 2024). Different footwork techniques exert varying biomechanical effects, and improper footwork can lead to abnormal stress on the knee joint, resulting in injury (Hua et al., 2024; Li et al., 2023). The Brush Knee and Twist Step is a typical forward step that necessitates coordination between the body’s center of gravity and the upper and lower limbs, significantly impacting biomechanics of the knee joint (Liu et al., 2024). Incorrect movement can cause abnormal stress distribution on the knee joint, increasing the risk of injury. Although existing research addresses the general health benefits of Tai Chi, there is a notable lack of systematic analysis regarding the specific biomechanical impact of particular footwork, such as the Brushing and Twisting Step, on the knee joint meniscus. This study analyzes the Brush Knee and Twist Step movement, exploring the contact stress characteristics of the knee joint meniscus during the motion, thereby providing a reference for developing more scientifically based exercise plans, reducing the risk of injury, and enhancing exercise safety.

## 2 Participants

### 2.1 Participant recruitment

The experimental protocols and procedures in this study were approved by the Ethics Committee of Fujian University of Traditional Chinese Medicine (approval number: 2023KS-81–1). Twenty healthy middle-aged and elderly male participants were recruited and stratified into two groups based on training duration: the Beginner Group (≤1 year, n = 10) and Experienced Group (≥5 years, n = 10). The inclusion criteria were as follows: 1) Healthy male individuals aged 45–65 years; 2) Absence of functional impairments or structural abnormalities in the knee or lower extremities; 3) Voluntary participation with signed informed consent. The exclusion criteria included: 1) A history of severe knee disorders; 2) Previous knee surgery; 3) A body mass index BMI≥30 kg/m^2^; 4) Systemic diseases affecting knee function (e.g., peripheral neuropathy, osteoporosis). The results of the basic examination are shown in Table 1.

**TABLE 1:** Statistical comparison of baseline characteristics between groups.

Parameter	(Mean ± SD)		*t*	*P*
	Beginner group	Experienced group		
Age (year)	58.40 ± 3.53	58.80 ± 3.85	0.242	0.812
Height (cm)	165.68 ± 3.98	166.63 ± 4.82	0.481	0.637
Weight (kg)	60.86 ± 2.97	61.73 ± 1.66	0.809	0.432
BMI(kg/m^2^)	22.29 ± 1.25	22.09 ± 1.49	−0.325	0.374

Note: *P < 0.05 indicates significant difference; **P < 0.01 indicates highly significant difference.

## 3 Methods

### 3.1 Kinematic measurement

Three-dimensional movement data during Tai Chi Knee Brushing and Twisting Step performance were collected using synchronized infrared motion capture (Oqus700+, Qualisys, Sweden) and force platforms (9260AA, Kistler, Switzerland). Ground reaction forces during Knee Brushing and Twisting Step execution were analyzed using Visual 3D biomechanical modeling software (Professional V6, C-Motion, United States).

### 3.2 Medical imaging measurement

Knee joint morphology in the supine extended-knee position was acquired using a LightSpeed 64-slice spiral CT scanner (GE Healthcare, United States) with scan coverage extending from the mid-to-distal femur to the proximal tibiofibular complex. The imaging parameters included a slice thickness of 0.625 mm and a resolution of 512 × 512 pixels. Three-dimensional MRI datasets of the same knee joint were obtained using a 3.0T MRI scanner (GE Healthcare, United States) with imaging parameters consisting of a slice thickness of 0.7 mm, an interslice gap of 0.5 mm, and a resolution of 512 × 512 pixels.

### 3.3 Finite element modeling

**FIGURE 1:**
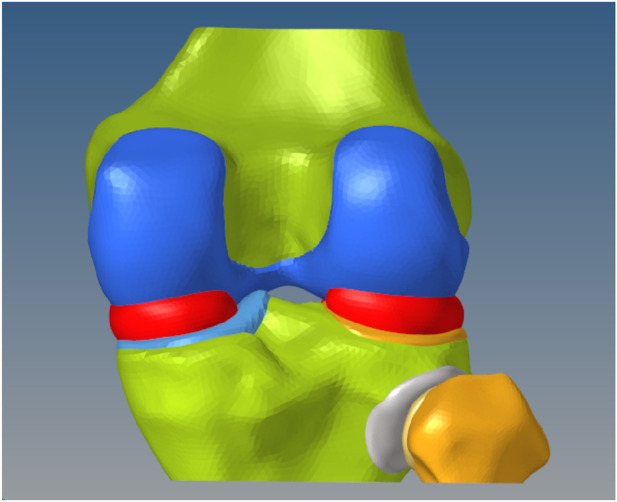
Knee joint finite element model.

Bone and cartilage structures were segmented from CT and MRI data using threshold-based techniques in Mimics (Materialise, Leuven, Belgium) and reconstructed into watertight solid 3D models. Surface patches were generated and converted into smooth solid models. A 2 mm inward offset operation created cancellous bone components, followed by Boolean operations to obtain cortical bone structures. Articular cartilage was integrated with osseous components to form the complete anatomical model (Figure 1).

### 3.4 Material parameter assignment

Mesh generation was conducted using HyperMesh (Altair Engineering, United States), resulting in an average of 225,100 elements and 58,900 nodes per model. Chondral and meniscal tissues were discretized with nearly incompressible hybrid elements (C3D4H), while osseous structures were meshed using standard tetrahedral elements (C3D4). Material properties were assigned based on established biomechanical literature (Table 2) (Bae et al., 2012; Dhaher et al., 2010; Segal et al., 2009).

**TABLE 2:** Material parameters.

Material	Elastic modulus (MPa)	Poisson’s ratio
Cortical Bone	17,000	0.3
Cancellous Bone	160	0.3
Meniscus	120	0.45
Articular Cartilage	10	0.45

### 3.5 Boundary constraints and loading protocol

**FIGURE 2:**
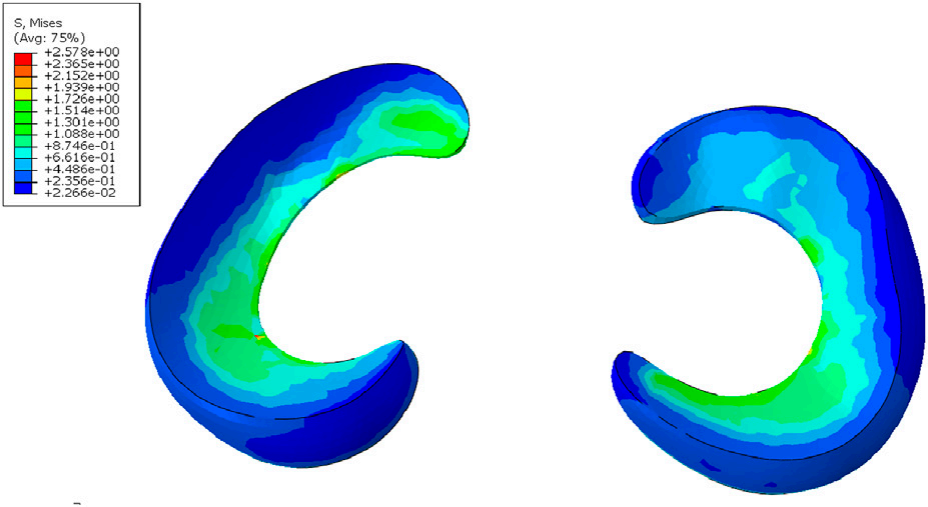
Stress validation of meniscus in model verification.

Interaction properties and loading conditions were defined within the Abaqus computational environment. TIE constraints were defined between cortical and cancellous bones, between cortical bone and cartilage, and between cortical bone and the meniscus. Frictionless contact was established between femoral cartilage and meniscus. The superior surface of the femur was kinematically coupled to a reference node, as concentrated loads must be applied to discrete nodal points through coupling operations. All translational and rotational degrees of freedom were fixed at the distal tibiofibular cross-sections, while axial compressive loading was applied along the anatomical axis of the femur through the proximal reference node. A vertical compressive load of 1000 N was applied to the proximal femur, resulting in medial and lateral meniscal stresses of 2.57 MPa and 2.37 MPa respectively, with model validity confirmed by previous biomechanical studies (Figure 2) (Bendjaballah et al., 1995; Keaveny and Hayes, 1993; Peña et al., 2006).

### 3.6 Finite element analysis

**FIGURE 3:**
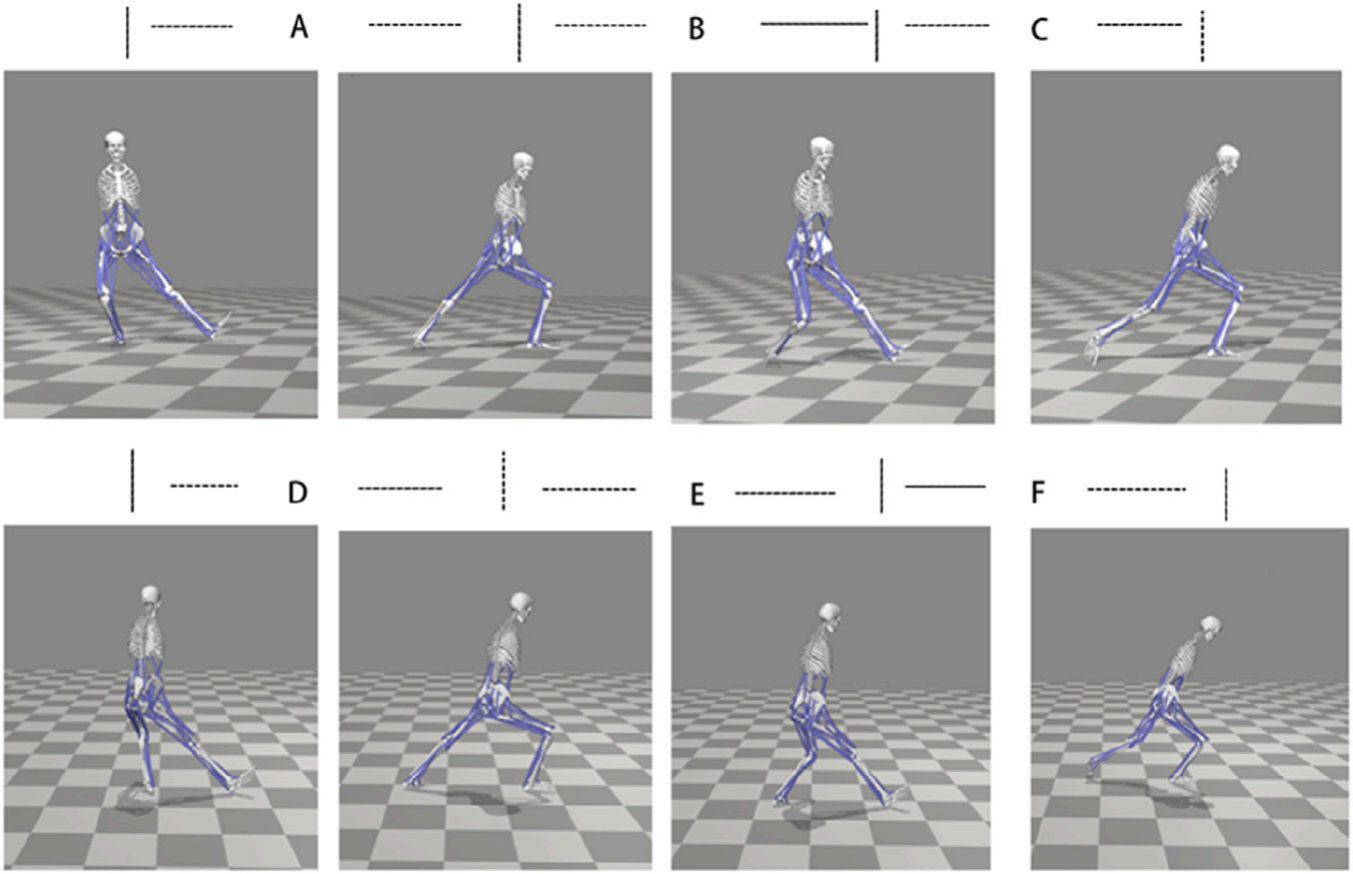
Movement Phases of Tai Chi. **(A)** (Left) Double-leg support phase **(B)** (Left) Swing phase **(C)** (Left) Single-leg support phase **(D)** (Right) Double-leg support phase **(E)** (Right) Swing phase **(F)** (Right) Single-leg support phase.

The boundary conditions and loading configurations for the finite element analysis were derived from kinematic and kinetic measurement analyses. The Knee Brushing and Twisting Step movement cycle was partitioned into six phases based on force platform recordings, bilateral movement symmetry, and established biomechanical criteria (Chang et al., 2021; Xu et al., 2003): left double-support phase, left swing phase, left single-support phase, right double-support phase, right swing phase, and right single-support phase (Figure 3).

A local coordinate system was established at the knee joint with the X -axis aligned with the femoral transepicondylar line, the Z -axis along the femoral longitudinal axis, and the Y -axis orthogonal to both. Joint angles obtained from Visual 3D analyses were utilized to manually refine the model’s angular configurations. During dynamic loading, the superior surface of the femur was kinematically coupled to a reference node, through which a downward axial force corresponding to the force platform recordings was applied along the anatomical axis.

### 3.7 Statistical analysis

To eliminate the influence of body weight on ground reaction forces, the ratio of ground reaction forces to body weight was used to represent dynamic data. Based on the bilateral symmetry and similarity of leg movements in Tai Chi, the biomechanical characteristics of the right leg were used to represent overall movement. The maximum and minimum angles of each joint were calculated to determine its range of motion. Statistical analysis was performed using SPSS 17.0 software (SPSS Inc., Chicago, IL, United States). The Shapiro-Wilk test was used to assess normality, and the results indicated that all parameter data were generally normally distributed. Assuming normality, an independent samples t-test was used to analyze the participants’ baseline characteristics. After ensuring overall normality and maintaining data independence through data matching, paired t-tests were used to statistically analyze the kinematic data of the hip, knee, and ankle joints in this study. This analysis evaluated the impact of different exercise levels on kinematic performance. All tests were two-tailed, and a p-value less than 0.05 was considered statistically significant. A p-value less than 0.01 indicated a highly significant difference.

## 4 Results

### 4.1 Kinematic results

**FIGURE 4:**
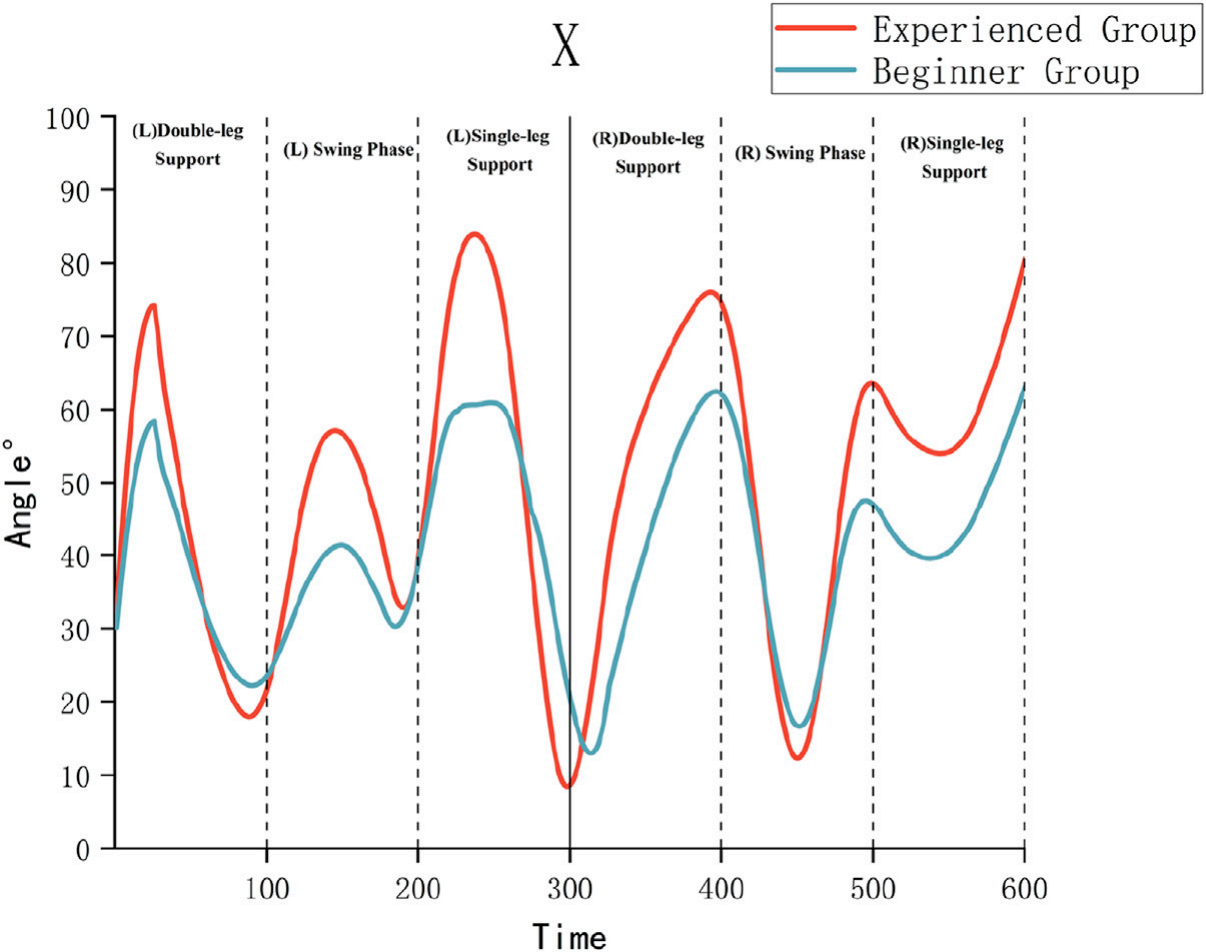
X-axis (flexion/extension) kinematic angles of the knee joint.

**FIGURE 5:**
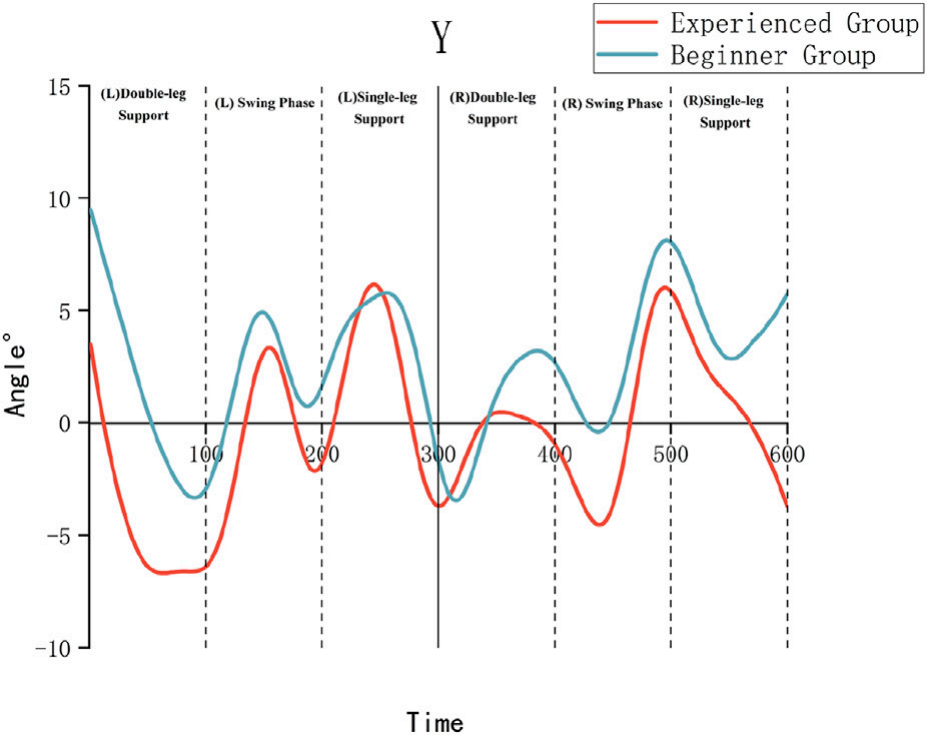
Y-axis (abduction/adduction) kinematic angles of the knee joint.

**FIGURE 6:**
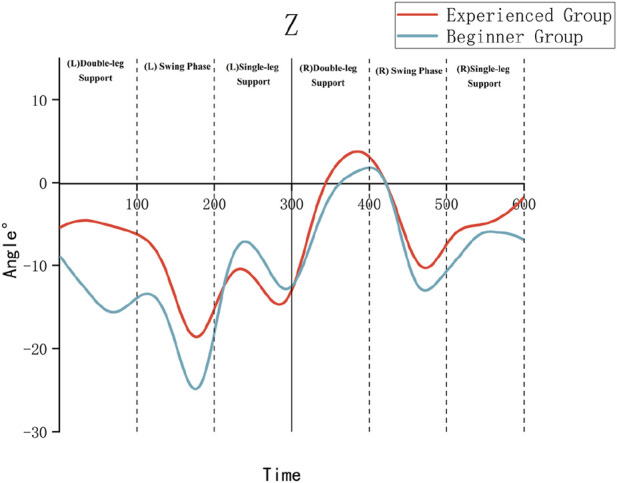
Z-axis (internal/external rotation) kinematic angles of the knee joint.

As illustrated in Table 3 and Figures 4–6, the Experienced Group exhibited significantly greater knee flexion angles throughout all movement phases compared to the Beginner Group (*P* < 0.01). For example, during the left swing phase, the Experienced Group displayed a knee flexion of 54.69° ± 4.05°, in contrast to 40.93° ± 5.44° in the Beginner Group. Additionally, the Experienced Group showed significantly smaller internal/external rotation angles, which indicates improved movement stability.

**TABLE 3:** Phase-specific knee joint biomechanical analysis.

Phase	Kinematic angle (°)	Beginner group (mean ± SD)	Experienced group (mean ± SD)	t	*P*
(L) Double-leg Support (L) Swing Phase (L) Single-leg Support (R) Double-leg Support (R) Swing Phase (R) Single-leg Support	X -axis	39.77 ± 14.27	45.11 ± 22.84	−6.205	<0.001**
	Y -axis	1.39 ± 4.14	−4.56 ± 2.90	31.126	<0.001**
	Z -axis	−13.51 ± 2.14	−5.07 ± 0.68	−42.641	<0.001**
	X -axis	34.68 ± 5.05	43.94 ± 10.7	−15.670	<0.001**
	Y -axis	1.65 ± 2.65	−0.91 ± 3.46	19.076	<0.001**
	Z-axis	−19.20 ± 4.97	−13.27 ± 4.88	−93.651	<0.001**
	X -axis	51.24 ± 10.38	56.00 ± 25.50	−3.086	0.030*
	*Y*-axis	4.25 ± 1.49	2.03 ± 3.42	9.823	<0.001**
	*Z*-axis	−9.94 ± 2.03	−12.43 ± 1.64	18.651	<0.001**
	X -axis	38.75 ± 18.57	53.45 ± 21.78	−24.436	<0.001**
	*Y*-axis	0.22 ± 2.96	−0.71 ± 1.5	4.818	<0.001**
	*Z*-axis	−3.32 ± 4.94	−1.16 ± 5.51	−26.322	<0.001**
	X -axis	36.96 ± 14.52	40.59 ± 20.74	−5.667	<0.001**
	*Y*-axis	2.91 ± 3.23	−0.39 ± 4.10	35.287	<0.001**
	*Z*-axis	−7.00 ± 5.97	−5.31 ± 4.83	−9.235	<0.001**
	X -axis	45.76 ± 6.72	60.70 ± 7.22	−117.932	<0.001**
	*Y*-axis	4.57 ± 1.52	1.06 ± 2.45	15.410	<0.001**
	*Z*-axis	−7.14 ± 1.60	−4.58 ± 1.27	−18.134	<0.001**

Note: *P < 0.05 indicates significant difference; **P < 0.01 indicates highly significant difference.

### 4.2 Dynamic results

**FIGURE 7:**
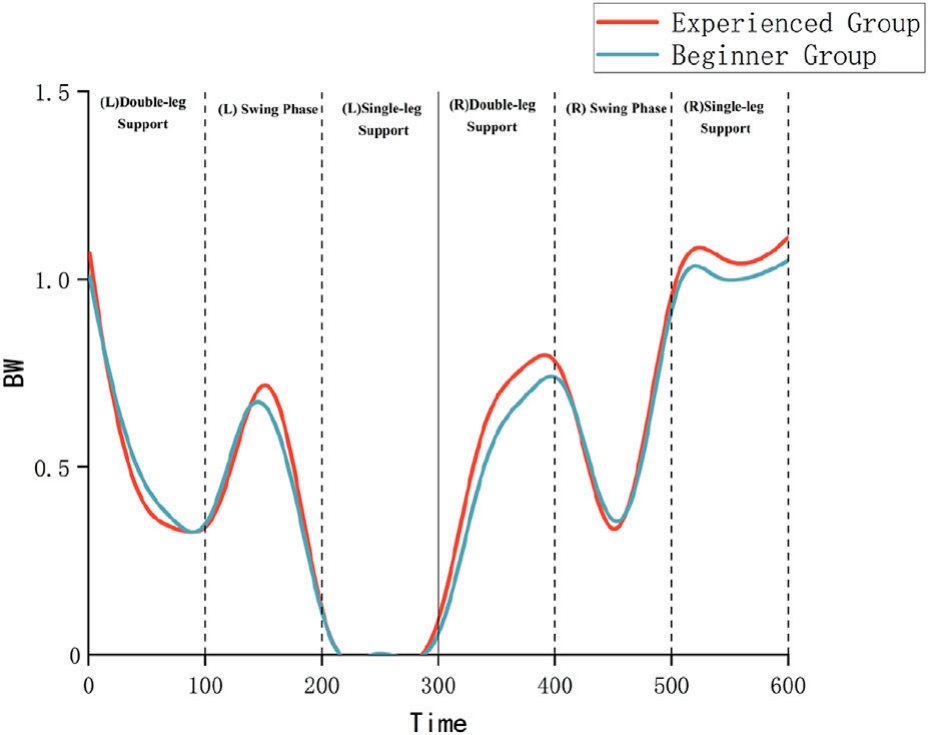
Ground reaction force (GRF) patterns across Tai Chi step-forward movement phases.

Both groups exhibited ground reaction forces (GRF) characterized by an initial ascending-descending trend. The experienced group demonstrated higher peak magnitudes and more uniform force distribution patterns compared to the novice group, as illustrated in Figure 7. For instance, during the (left) double-support phase, the experienced group exhibited a GRF peak of 1.07 times body weight (BW), whereas the novice group demonstrated a peak of 1.02 BW. During the (right) single-support phase, the experienced group displayed smoother GRF profiles, indicative of superior neuromuscular control capabilities.

### 4.3 Loading phase selection and contact stress outcomes

#### 4.3.1 Temporal load application criteria

Computational evaluations were conducted at characteristic time nodes that correspond to local peaks or significant kinematic and kinetic transitions during various phases of Knee Brushing and Twisting Step, This analysis revealed a 9% variation for beginners and an 8% variation for experienced practitioners in the selected phases, with detailed data presented in Table 4.

**TABLE 4:** Loading data across.

Phase	Group	Time point	X -axis	Y -axis	Z -axis	Load
(L) Double-leg Support	BG	9%	59.07°	8.7°	−10.29°	1.01
	EG	8%	57.48°	7.74°	−8.35°	0.85
(L)Swing Phase	BG	52%	40.93°	5.44°	−1.33°	0.7
	EG	52%	54.69°	4.05°	−6.42°	0.68
(R)Double-leg Support	BG	97%	62.39°	3.68°	−0.44°	0.74
	EG	8%	57.48°	7.74°	−8.35°	0.85
(R)Swing Phase	BG	89%	43.18°	8.48°	−12.51°	0.75
	EG	52%	54.69°	4.05°	−6.42°	0.68
(R)Single-leg Support	BG	99%	61.59°	5.69°	−7.16°	1.05
	EG	99%	78.51°	−3.93°	−3.15°	1.09

#### 4.3.2 Contact Stress Results

## 5 Discussion

Tai Chi is a low-impact, slow, and continuous exercise that exerts less contact stress on the body compared to walking and running (Li et al., 2019). Research indicates that an appropriate biomechanical environment and moderate pressure stimulation are essential for maintaining joint cartilage health, while prolonged overload can lead to cartilage degeneration (Griffin and Guilak, 2005; Louboutin et al., 2009; Morimoto et al., 2009; Sah et al., 1989; Waldman et al., 2004). Therefore, engaging in rational exercise and avoiding excessive load are vital for the prevention and treatment of joint diseases.

This study integrates kinematic and dynamic experiments with finite element simulations to investigate the biomechanical effects of the Brush Knee and Twist Step on the knee joint meniscus in middle-aged and elderly individuals. The results revealed that the Experienced Group exhibited superior knee flexion angles, ground reaction force distribution, and meniscal contact stress distribution compared to the Beginner Group, resulting in more stable movements and a more evenly distributed contact stress. Additionally, the Beginner Group demonstrated stress concentration on the lateral meniscus during most phases, which may increase the risk of injury (Table 5). This phenomenon can be attributed to a lack of coordinated movement between the hip, knee, and ankle joints during Tai Chi practice. Such miscoordination alters femoral-tibial contact positions, lead to uneven stress distribution, and increases the burden on the knee joint, resulting in excessive wear or injury to the joint surfaces.

**TABLE 5:** Contact stress results.

Phase	Group	Time point	Contact stress and location
(L) Double-leg Support	BG	9%	4.6 MPa,ALM
	EG	8%	4.2 MPa,ALM
(L)Swing Phase	BG	52%	4.6 MPa,ALM
	EG	52%	4.5 MPa,ALM
(R)Double-leg Support	BG	97%	4.7 MPa,MP-LM
	EG	8%	4.5 MPa,ALM
(R)Swing Phase	BG	89%	4.4 MPa,ALM
	EG	52%	4.5 MPa,AMM
(R)Single-leg Support	BG	99%	4.5 MPa,ALM
	EG	99%	4.6 MPa,PMM

Related studies have shown that a lower knee posture during Tai Chi correlates with an increased load on the joint (Wen et al., 2018). However, the magnitude of stress on articular cartilage is associated with both the overall load on the joint and the distribution of this load across the cartilage contact area (Ahmed et al., 1983). In this study, although the Experienced Group maintained a lower posture, their peak contact stress values did not differ significantly from those of the Beginner Group. Additionally, the stress distribution patterns in the Experienced Group were significantly superior, indicating more efficient load management.

On the other hand, increased internal and external rotation angles of the knee joint significantly elevate cartilage stress (Liao et al., 2015), potentially leading to heightened strain on the surrounding ligaments (Lee et al., 2001). Furthermore, excessive internal or external tilt can result in increased stress. Biomechanical studies suggest that when the tilt angle is between 6° internal and 10°external (Zhang et al., 2024), the stress variations on the medial and lateral sides of the knee joint are not significant. In this study, both the Beginner and Experienced Groups exhibited internal or external tilt angles within the range of −6°–10°, indicating that the internal or external tilt of the knee joint did not result in notable stress variations.

**FIGURE 8:**
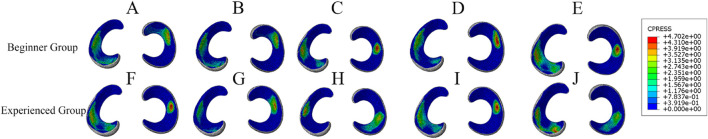
Contact Stress Results. Note: In the Beginner Group, (left) double-support phase at 9% **(A)**, (left) swing phase at 52% **(B)**, (right) double-support phase at 97% **(C)**, (right) swing phase at 89% **(D)**, and (right) single-support phase at 99% **(E)**. For the Experienced Group, (left) double-support phase at 8% **(F)**, (left) swing phase at 52% **(G)**, (right) double-support phase at 93% **(H)**, (right) swing phase at 88% **(I)**, and (right) single-support phase at 99% **(J)**.

Moreover, Tai Chi footwork emphasizes the concept of changing between full and empty, where certain parts of the body must remain relaxed (empty) while others generate force or bear weight (full). During lower limb movements, one leg (the full leg) supports most of the weight and force, while the other leg (the empty leg) remains relatively relaxed, prepared to move or generate force. By shifting the body’s center of gravity, Tai Chi achieves coordination between internal and external movements, resulting in a light and stable effect. Yang Chengfu (Yang, 2005) in his ‘Treatise on Tai Chi Practice’ emphasized that ‘Tai Chi practice begins with the distinction between full and empty; only when this distinction is made can one rotate lightly and effortlessly. If this distinction is overlooked, steps become heavy and unstable.’ During the swing phases of the left and right legs, the Experienced Group exhibited a relatively greater knee flexion angle and bore a greater load, which may increase contact stress and affect the menisci and cartilage. However, through outward swinging and shifting the center of gravity to the opposite side, stress concentration was effectively distributed, thereby reducing the load on a single knee joint. During different stages of the knee-hugging and stepping exercise, the contact stress on the knee joint at peak moments showed a more uniform distribution for the long-term practice group, while the contact stress for the novice group was concentrated in the lateral meniscus (Figure 8). This further illustrates the role of combining emptiness and fullness in Tai Chi practice. The integration of ‘full and empty’ prevents the knee joint from enduring excessive continuous pressure and helps mitigate cartilage wear. However, if the ankle joint’s power control and direction are not precise during the center of gravity transfer, it may affect the knee joint’s motion angle, leading to unnecessary stress concentration and increasing the risk of injury. The results show that the beginner group had higher internal or external rotation angles than the Experienced Group, indicating that the beginner group relied more on joint motion than on muscle control to maintain stability. The Experienced Group, despite having greater knee flexion, exhibited more even contact stress distribution, suggesting better knee joint muscle strength and control ability, allowing them to perform lower postures more safely. This is a key factor in enhancing leg strength and stability in Tai Chi practice.

Therefore, when practicing the advancing movement, it is crucial to focus on the technique of ‘changing between full and empty.’ This ensures the correct distribution of the body’s center of gravity, which protects the knee joint and maximize the benefits of Tai Chi practice. During the process of transferring the center of gravity, it is also essential to emphasize the coordination of the lower limb joints should. Utilizing muscle control to stabilize the joints is important to avoid over-reliance on knee joint motion.

This study indicates that long-term Tai Chi practice enhances the balance of meniscal stress distribution in the knee joint, reducing the risk of injury. It provides theoretical support for the scientific practice of Tai Chi among middle-aged and elderly individuals. By understanding and applying Tai Chi principles such as clear differentiation between full and empty, flexible transition, and coordinated movement, practitioners can achieve better coordination of the lower limb joints, leading to a more even distribution of stress across the knee joint. Through continuous practice, this coordination gradually becomes a natural state of the body, fostering beneficial movement pattern that enable practitioners to utilize muscle control for stabilizing the joints. This reduces unnecessary joint motion and minimizes knee wear and injury, ultimately promoting joint health.

However, this study has certain limitations. First, the sample size was restricted to male participants aged 45–65 years. Future research should expand the sample size to include female participants and other age groups. Second, this study focused solely on the dynamic characteristics of the Brush Knee and Twist Step movement. Future studies could investigate the biomechanical impact of other Tai Chi movements on the knee joint. Furthermore, the finite element model was constructed based on individual imaging data, and despite validation, its complexity limited the simulation of certain real-life biomechanical environments. Future research could incorporate more advanced dynamic imaging technologies to enhance the model’s accuracy and practical application.

## Data Availability

The original contributions presented in the study are included in the article/supplementary material, further inquiries can be directed to the corresponding author.
